# Supplementary material for: Effects of the Replacement of Dietary Fish Meal with Poultry By-Product Meal on Growth and Intestinal Health of Chinese Soft-Shelled Turtle (Pelodiscus sinensis)
Source: Animals (Basel). 2023 Feb 27;13(5):865. doi: 10.3390/ani13050865 (PMC10000163; doi:10.3390/ani13050865)
Supplement: Supplementary file 1 [file animals-13-00865-s001.zip › animals-2201311-supplementary.pdf]

Table S1. Chemical composition of the fish meal and poultry by-product meal.

	**Fish meal**	**Poultry by-product meal**
Dry Matte %	95.66	94.03
Crude protein %	63.73	63.16
Crude lipid %	8.98	10.24
Ash %	15.09	12.40
**EAA[1] g/100g**		
Arg	4.06	4.06
Cys	0.48	0.59
His	1.46	1.26
I1e	2.58	2.37
Leu	4.35	4.77
Lys	4.66	3.65
Met	1.81	1.22
Phe	2.58	2.16
Tyr	1.84	1.85
Thr	2.60	2.18
Trp	0.70	0.80
Val	2.98	2.53
**NEAA[2] g/100g**		
Ala	4.19	4.01
Asp	5.29	4.60
Glu	8.21	7.31
Gly	5.58	5.36
Pro	3.44	3.72
Ser	2.00	2.08

[1]EAA, essential amino acid.

[2] NEAA, non-essential amino acids.

Table S2. Ingredients composition of the experimental diets (%).

Ingredients	Dietary Treatment[1]			
	**PBM0**	**PBM5**	**PBM10**	**PBM15**
Fish meal	55.00	50.00	45.00	40.00
Poultry by-product meal	0.00	5.00	10.00	15.00
Soy protein concentrate	5.00	5.00	5.00	5.00
Wheat meal	25.80	25.80	25.80	25.80
Soybean oil	3.00	3.00	3.00	3.00
Extruded soybean	5.00	5.00	5.00	5.00
Shrimp paste	3.00	3.00	3.00	3.00
Choline chloride	2.00	2.00	2.00	2.00
Vitamin premix[2]	0.20	0.20	0.20	0.20
Mineral premix[3]	0.50	0.50	0.50	0.50
Choline chloride	0.50	0.50	0.50	0.50

[1]PBM0, PBM5, PBM10 and PBM15 (no replacement of fish meal, PBM replacement of 5%, 10%, 15% of fish meal). The same below

[2]Vitamin premix: provided by Zhejiang Jindadi Agricultural Co., Ltd., Zhejiang, China.

[3]Mineral premix: provided by Zhejiang Jindadi Agricultural Co., Ltd., Zhejiang, China.

Table S3. Chemical and amino acid composition and fatty acid profile of the test diets.

	**Dietary Treatment[1]**			
	Con	PBM5	PBM10	PBM15
**Proximate composition % as fed**				
Dry matter	90.65	90.88	90.59	90.74
Crude protein	43.89	43.86	43.83	43.80
Crude lipid	9.73	9.79	9.86	9.92
Crude ash	12.60	12.47	12.33	12.20
**EAA[1] g/100g**				
Arg	2.89	2.89	2.89	2.89
His	1.07	1.06	1.05	1.04
I1e	1.86	1.85	1.84	1.83
Leu	3.16	3.19	3.21	3.23
Lys	3.14	3.09	3.03	2.98
Met	1.15	1.13	1.10	1.07
Cys	0.44	0.44	0.45	0.46
Phe	1.92	1.90	1.88	1.85
Tyr	1.35	1.35	1.35	1.35
Thr	1.82	1.80	1.78	1.76
Trp	0.44	0.44	0.45	0.45
Val	2.13	2.11	2.09	2.07
**NEAA[2] g/100g**				
Ala	2.83	2.82	2.81	2.80
Asp	3.94	3.91	3.87	3.84
Glu	6.50	6.45	6.41	6.36
Gly	3.59	3.58	3.57	3.55
Pro	2.58	2.59	2.61	2.62
Ser	1.63	1.63	1.64	1.64

[1]PBM0, PBM5, PBM10 and PBM15 (no replacement of fish meal, PBM replacement of 5%, 10%, 15% of fish meal). The same below

[2]EAA, essential amino acid.

[3] NEAA, non-essential amino acids.

Table S4. Primer sequences of the intestinal and liver genes of the Chinese soft-shelled turtle.

Gene	Accession Number	Pair primers	Size(bp)	Tm(°C)
β-actin	XM_006134860	F:TGAGCTTCGTGTAGCACCTG<br>R:AGGATGGCATGGGGTAAAGC	252	58.18<br>58.02
IL-10	KT203380	F:ACAGGAAATATGGGGAAGGACG<br>R:AAGATTTAAACTGAGGTTCTGGAAG	126	56.79<br>52.13
IFN-γ	JN021380	F:GTCCCAACCAACGGCAAAC<br>R:GACTTTGTTGCTTCAAACGGG	195	57.87<br>54.85
TNF-α	XM_014575959	F:TCCTCCGGCACATCATCTTG<br>R:GTACCACACTTCGGTCTCGG	116	57.51<br>58.18
IL-1β	NM_001317048	F:TCCAACACCAAGTGAGGCTG<br>R:ACTCAAACTGGGTGGTGTCC	249	57.90<br>57.64
IL-8	FJ472848	F:AGTGAGTTGTTTGTCCTCAATTAGT<br>R:AGCTTGGGGCAGAGAAGAGA	209	53.47<br>58.53
IL-15	XM_006121622	F:ACATACGTGAAGATGAATGTGAAGT<br>R:GCGCACATGCTGTTGGATTT	116	53.51<br>57.07
IGF-1	NM_001286920	F:CAAGCCACCCAAATCTGCAC<br>R:CCTGTGTTCCCTCGACTTGT	105	57.55<br>57.30
TLR4	NM_001286933	F:ATTGCGTCCCAGTGTACGTT<br>R:AGGGACTTGAATGTGCTCGG	176	56.88<br>57.71
TLR5	XM_025180981	F:ACCATCTACTCTCCGTTGCC<br>R:GATCCCTCGGTACTCTCCCA	185	56.92<br>58.43
TLR8	XM_014573456	F:TGCTATCTGCTCCTGTGGTG<br>R:GGGATTCATGTTGCCTTTCTTT	106	57.21<br>53.23

β-actin: Beta-actin; *IL-10*: Interleukin-10; *IFN-γ*: interferon-γ *TNF-α*: Tumor necrosis factor-*α; IL-1β*: Interleukin-1β; *IL-8*: Interleukin-8; *IL-15*: Interleukin-15; *IGF-1*: Insulin-like growth factor 1; *TLR4*: Toll-likereceptor4; *TLR5*: Toll-likereceptor5; *TLR8*: Toll-likereceptor8;
